# Comparing between results and complications of doing voiding cystourethrogram in the first week following urinary tract infection and in 2-6 weeks after urinary tract infection in children referring to a teaching hospital

**DOI:** 10.15171/jrip.2016.30

**Published:** 2016-04-09

**Authors:** Parsa Yousefichaijan, Fatemeh Dorreh, Someyeh Shahsavari, Abdolghader Pakniyat

**Affiliations:** ^1^Amirkabir Hospital, Department of Pediatric, School of Medicine, Arak University of Medical Sciences, Arak, Iran; ^2^Department of Emergency Medicine, Arak University of Medical Sciences, Arak, Iran

**Keywords:** Urethritis, Pyelonephritis reflux, Urinary tract infection, Voiding cystourethrogram

## Abstract

**Introduction:** Urinary tract infection is the most common genitourinary disease in children so about 40% of the children with urinary tract infection suffering from reflux that caused some consequences such as pyelonephritis and kidney parenchymal injury.

**Objectives:** This research was conducted to compare the timing of voiding cystourethrogram (VCUG) in children with urinary tract infection in first week and after the first week of urinary tract infection.

**Patients and Methods:** This research is a case-control study that both case and control groups include 208 children from 1 month to 12 years old with the complain of urinary tract infection. In case group, the VCUG was performed at the first week of infection and in control group, the VCUG was performed after the first week of infection.

**Results:** complication such as dysuria was observed in two-thirds of children who VCUG was performed during first week after urinary tract infection. Parents stress in case group was more than the other (*P*=0.015). For overall, the incidence of reflux in case and control groups was 49.5% and 50%, respectively. The mean of reflux grading in right kidney in case group was lower than control group resulting in significant differences between two groups.

**Conclusion:** According to higher grade of stress in parents and complications due to VCUG at the first week of urinary tract infection, it is suggested that VCUG be conducted on selective patients in the hospital at the first week of urinary tract infection and during hospitalization.

**Implication for health policy/practice/research/medical education:**

Performing VCUG at first week of urinary tract infection will not lead to an increase in the prevalence of reflux in these patients.

## Introduction

Urinary infection is a common problem in children, which occurs 3-5% in girls and 1% in boys (1-4). Reflux is the cause of urinary tract infection which is a congenital disease due to ureterovesical junction deficit, to diagnose the reflux, voiding cystourethrogram (VCUG) is used but there is not a constant idea when the VCUG should be done (3-7). Based on pediatric urology textbooks, VCUG method is proposed to be done 4 to 6 weeks after treatment has been started, since urinary tract infection with ureterovesical inflammation can lead to temporary reflux or aggravate the present reflux (1,8,9). However, others believe that VCUG must be done as soon as possible (10-12). Moreover, acute infection can cause urethral dilation and thus increase reflux grade. If this fact be considered right, the prevalence of reflux would be higher, when VCUG is done in the first week after urinary infection, compared to further time. If VCUG is performed later, patients have to take prophylaxis antibiotic and families will be anxious about deterministic diagnosis. Additionally, some patients might not follow up and do not come back after they are discharged from the hospital.

## Objectives

This study is intended to get to is there a relationship between the time of VCUG and reflux prevalence and its severity or not?

## Patients and Methods

In an analytic-observational (case–control) study 208 patients (1 month to 12 years old), complaining urinary tract infection with indication of VCUG were surveyed about the time they do VCUG after parents’ justification and their satisfaction in Amir-Kabir hospital from 2005 through 2007.

In case group, VCUG was conducted during first week fallowing urinary tract infection. In control group, VCUG was conducted after the first week (when urine culture result was negative). In this study, all patients were treated with antibiotics (prophylaxis). All VCUG results were reported by a single radiologist. Grading was performed according to *Nelson Pediatric Textbook* and radiologist was not aware of the study and did not know to which group they belong. All patients were surveyed about catheterization complaints like pain and dysuria with questionnaire for 7 days. Parental stress inventory consisted of four score codes. Code 1 means no parental stress, code 2 means little parental stress, code 3 means moderate parental stress and code 4 means severe parental stress after their child have undergone VCUG. Then paternal performance and stress was surveyed and comparison between two groups was done.

The characteristic to enter the study included all under five kids with one period of urinary tract infection with hyperthermia or any girl in the school period with two or more period of urinary infection. It also included any boy with urinary infection. The cases were excluded if parents did not accept to take VCUG or if secondary reflux (caused by neurogenic bladder) had be seen in their VCUG.

### Ethical issues

The research followed the tenets of the Declaration of Helsinki. Informed consent was obtained. This study was approved by the Ethics Committee of Arak University of Medical Sciences.

### Statistical analysis

All variables were analyzed by SPSS 16 software. Descriptive statistical methods (frequency, percentage and mean ± SD) were used for the statistical evaluation. Mann-Whitney U, chi-square and NPAR tests were used for analytical evaluation. *P* value less than 0.05 was considered significant in the study.

## Results

VCUG was conducted in 208 patients in two groups, each include 104 patients. Case group included 17 boys (15.4% of society) and 87 girls (84.6% of society) with average age of 46 moths and control group included 21 boys (19.4% of society) and 83 girls (80.6% of society) with average age of 41 moths. Statistically no significant difference was observed between case and control groups (*P*=0.46).

In case group, reflux rate in right and left kidney in observed kids were 31.1% and 36.5% respectively. In control group, reflux rate in right and left kidney in observed kids were 41.3% and 38.5% respectively. Statistically no significant difference was observed between case and control groups (*P*=0.14). Furthermore, in case and control groups, no statistical difference was seen (*P*=0.88; Tables 1 and 2).

**Table 1:** Reflux severity determination in right and left kidney in kids 1 month to 12 years

	**Group grading**						**Total**
	**Grade I**	**Grade II**	**Grade III**	**Grade IV**	**Grade V**	**No reflux**	
Case	Right kidney	%	2.9	9.6	15.4	1.9	‏1	69.2	‏100
		n	‏3	‏10	‏16	‏2	‏1	‏72	‏104
	Left kidney	%	2.9	10.6	12.5	7.7	2.9	63.4	‏100
		n	‏3	‏11	‏13	‏8	‏3	‏66	‏104
	Both kidney	%	5.8	20.2	27.9	9.6	3.9	132.6	‏200
		n	‏6	‏21	‏29	‏10	‏4	‏138	‏208
Control	Right kidney	%	1.9	8.7	20.1	5.8	4.8	58.7	‏100
		n	‏2	‏9	‏21	‏6	‏5	‏61	‏104
	Left kidney	%	3.9	5.8	18.2	6.7	3.9	61.5	‏100
		n	‏4	‏6	‏19	‏7	‏4	‏64	‏104
	Both kidneys	%	5.8	14.5	38.3	12.5	8.7	120.2	‏200
		n	6	‏15	‏40	‏13	‏9	‏125	‏208

**Table 2:** Reflux severity determination in right and left kidney of kids 1 month to 12 years old

**Kidney**	**Group**	**Reflux severity**	***P*** ** value**
Right	Case	2.6 (2.3-2.9)	0.045
	Control	3.07 (2.7-3.3)	
Left	Case	2.9 (2.5-3.2)	0.604
	Control	3.05 (2.6-3.4)	

In this study, overall reflux prevalence in kids of one month to 12 years old, in case and control groups, were 49.5% and 50% respectively (*P*=0.94).

In this study, patients were surveyed on catheterization complications that VCUG complications were significantly lower in the group 2 to 6 weeks (*P*=0.001). Additionally parental stress level of these group was lower (*P*=0.015).

## Discussion

In this analytical case-control study, VCUG was done on 208 girl and boy patients in two groups, each included 104 patients. No significant difference was observed between two groups according to age and gender. Also, no significant difference in the prevalence of right and left kidney refluxes was detected. No significant difference in overall reflux prevalence between two groups was observed. The data of this study was in accordance with pervious researches. Mahant et al revealed that timing of VCUG did not influence the prevalence and severity of reflux during the first week and later (7). Results of other studies were in accordance with report of Mahant et al. They recommended that VCUG should be conducted as soon as possible (6,10-13). Previous authors believes that VCUG should be done at the end of treatment, when discharging the patient from hospital (1). Delaying the schedule of VCUG from yearly to every 2 years in children with mild VUR and every three years in children with moderate/severe vesicoureteric reflux yields substantial reductions in the average numbers of VCUGs and costs, with a modest subsequent increase in x-ray exposure (12). The rate of detection of VUR in children with a first episode of UTI does not increase when the VCUG is done early (within the first 7 days of diagnosis) rather than later (13).

This study also supported the results of a study which was conducted in Royal hospital in Sydney of Australia. In this study Craig et al investigated 272 patients, only 2 patients underwent VCUG in the first week and all others took VCUG later. No significant relationship between the time of VCUG and reflux severity and prevalence was detected. However according to low proportion of patients who had taken VCUG in the first week, no comparison could be done on reflux severity and prevalence (11). On contrary, in the current study the proportion of cases and control group are equal hence, comparison is logical and reasonable.

Mean reflux severity in left kidney of two groups did not show significant difference, while in the right kidney mean reflux severity of control group was more than case group and showed significant difference.

Regarding the complications it should be noted that two-thirds of kids who had taken VCUG in the first week experienced dysuria. However kids who take VCUG later did not report any complications which led to significant difference.

## Conclusion

Since prevalence and severity of reflux does not show difference in case and control groups, it is recommended that selected patients, who might not refer for VCUG later, take VCUG in the first hospitalization. Also, since complications of kids who had taken VCUG in week 2 to 6 after urinary infection, were far less, and to reduce parental anxiety and psychological injury, it is recommended that VCUG be done 2 to 6 weeks after urinary infection.

## Limitation of the study

Our study including a relatively small sample size and absence of long-term follow up.

## Acknowledgments

The research team wish to thank vice chancellor of research for their financial support and also children and their parents who contribute to this research.

## Authors’ contribution

All authors contributed to design of the research. PY, FD and SS conducted the research. SS and AP analyzed the data. PS and AP prepared the manuscript. All authors read, revised, and approved the final manuscript.

## Conflicts of interest

The authors declared no competing interests.

## Ethical considerations

Ethical issues (including plagiarism, misconduct, data fabrication, falsification, double publication or submission, redundancy) have been completely observed by the authors.

## Funding/Support

The mentioned findings in this essay has been excerpted from MD thesis of Dr. Somayeh Shahsavari under in Arak, which has been conducted with sponsorship from Arak University of Medical Sciences (research project/grant # Behsan-314).
